# Effect of ouabain on myocardial remodeling in rats

**DOI:** 10.3892/etm.2013.1098

**Published:** 2013-05-02

**Authors:** YANPING REN, MINGJUAN ZHANG, TING ZHANG, RUOWEN HUANG

**Affiliations:** 1 Geriatric-Cardiovascular Department, The First Affiliated Hospital, Medical College of Xi’an Jiaotong University, Xi’an, Shaanxi 710061;; 2 Cardiovascular Department, The Second Affiliated Hospital, Medical College of Xi’an Jiaotong University, Xi’an, Shaanxi 710003, P.R. China

**Keywords:** ouabain, myocardic, remodeling

## Abstract

The aim of this study was to investigate the effect of ouabain (EO) on myocardial remodeling. Twenty-two adult male Sprague-Dawley rats were randomly divided into two groups: the rats in the EO group (n=12) were intraperitoneally injected with EO daily and those in the control group (n=10) were injected with physiological saline daily. After 8 weeks the rats were sacrificed. The ultrastructural changes in the myocardium were observed. The expression levels of voltage-gated potassium channel 4.2 (K_V_4.2) were detected by real-time quantitative reverse transcription-polymerase chain reaction. The effects of EO on the myocardial action potential and transient potassium efflux (I_to_) were measured by patch clamping. The systolic blood pressure (SBP) of 10 of the 12 rats in the EO group, designated as the EO-sensitive (OS) rats, began to increase from the fifth week of treatment and was significantly higher compared with that of the control group 6 weeks later (P<0.01). The remaining 2 rats in the EO group that presented no increase in SBP following 8 weeks of treatment (P>0.05) were designated as EO-resistant (OR) rats. Pathological ultrastructural changes were significant in the apical mid-myocardium of the OS rats. No significant differences in K_V_4.2 expression were observed among the OS, OR and control rats. The patch clamp results revealed that EO prolongs the action potential duration, reduces I_to_ and triggers the electrical remodeling of the myocardium. EO induces a blood pressure increase and triggers structural and electrical remodeling.

## Introduction

Sodium/potassium pump inhibitors in human plasma are identical or similar in structure to plant-derived ouabain (EO) and are synthesized by the adrenal cortex (1). Since Hamlyn *et al* confirmed that EO was an endogenous sodium pump inhibitor in 1991 (2), it has been gradually elucidated as an important factor that participates in the pathogenesis of hypertension in the neuroendocrine network. The persistent and specific association of EO with hypertension indicates that 40–50% of patients with mild to moderate primary hypertension have elevated EO, with normal dietary salt intakes (3–6). Evidence indicates that EO does not fulfill the criteria for a putative natriuretic hormone; however, it is essential in the adaptation to sodium depletion and sodium loading (7). Several lines of experimental evidence clearly demonstrate the prohypertensive role of EO, including induction of hypertension in EO-treated rodents, elevation of EO levels in hypertensive rats and observations of the central prohypertensive action of this hormone. Three mechanisms for the prohypertensive effect, including the ‘adducin paradigm’, the existence of highly sensitive EO-binding sites in vascular smooth muscle and central effects, were proposed to link EO to vasoconstriction in hypertension (8). Chronic EO treatment produces hypertension, which depends on the activation of central nervous mechanisms associated with increased sympathetic tone, subsequent to the activation of the brain renin-angiotensin (9) and endothelin systems (10), as well as peripheral vascular mechanisms (11). In addition, vascular endothelial cells, whose functional integrity is crucial for the maintenance of blood flow and antithrombotic activity, may be a target for endogenous EO. We previously studied the effect of EO on human umbilical vein endothelial cells (HUVECs) and identified that EO stimulates the proliferation of HUVECs at physiological concentrations (0.3–0.9 nmol/l). However, cell death was induced at pathological concentrations (0.9–1.8 nmol/l), including the swelling phenomenon and the appearance of apoptotic bodies (12). Based on our previous study, the effects of EO on endothelial dysfunction and cardiac remodeling were investigated in EO-sensitive (OS) hypertensive rats.

## Materials and methods

### Animal model

A total of 22 adult male Sprague-Dawley rats, weighing 180–250 g, SPF grade, were provided by the Laboratory Animal Center of Xi’an Jiaotong University College of Medicine. The local legislation for ethics on experiments on animals and guidelines for the care and use of laboratory animals (from the Ethics Committee of Xi’an Jiaotong University) were followed in all animal procedures. All animals were allowed time to adapt to the laboratory environment with free access to food and water in temperature- and humidity-controlled housing with natural illumination for a week and subsequently fasted for 12 h prior to the experiment. The animals were randomly divided into two groups: the EO group (O group, n=12) and the control group (N group, n=10). The O group was treated with 20 *μ*g/kg/day EO by intraperitoneal injection for 8 weeks. The N group was treated with 1 ml/kg/day normal saline by intraperitoneal injection for 8 weeks. Weight and systolic blood pressure (SBP) were recorded three times for one week and the mean values were calculated. SBP was measured via an indirect tail cuff method using a blood pressure recorder for rats (RBP-1B; China-Japan Clinical Medicine Institute, Beijing, China). After 6 weeks, the O group was divided into two groups based on their recorded BPs: the OS group (BP increased, n=10) and the EO-resistant (OR) group (no clear increase in BP, n=2).

### Ultrastructural changes in the myocardium

The rats were anesthetized with a bolus dose of 20% urethane (6 ml/kg) and fixed on the surgery table. Following the exposure of the heart through rapid thoracotomy, the middle left ventricular free wall of the myocardium and the proximal end of the ascending aorta were collected. The abdominal cavity was opened and the kidney cortex was removed. The tissue specimens were cut into pieces (1×1×1 mm) and washed in phosphate buffer (pH 7.6). Glutaraldehyde fixation solution was used to postfix the tissue, which was then dehydrated in graded concentrations of acetone. The tissues were embedded in Araldite, cut into thin sections, stained with uranyl acetate and lead citrate and examined under a Hitachi H-600 transmission electron microscope (Hitachi, Tokyo, Japan).

### Real-time quantitative reverse transcription-polymerase chain reaction (RT-PCR)

Total RNA was extracted from the rat myocardial tissue using TRIzol reagent according to the manufacturer’s instructions (Takara Bio Inc., Shiga, Japan). The quality of the total RNA was determined spec-trophotometrically at 260 and 280 nm. Following agarose gel electrophoresis, cDNA was synthesized using M-MLV reverse transcriptase according to the instructions of the manufacturer (Takara Bio Inc.). All cDNA samples were used immediately or stored at −20°C until analysis.

Primers and probes were designed and synthesized by Sangon Biotechnology Co., Ltd. (Shanghai, China). Glyceraldehyde-3-phosphate dehydrogenase was used as the control. The primer and probe sequences are listed in Table I.

The K_V_4.2 mRNA expression levels in the rat myocardial tissue in the different groups were detected by RT-PCR. The reaction mixture contained 1 *μ*l each forward and reverse primers, 1 *μ*l fluorescent probe, 1 *μ*l RT/platinum^®^ Taq mix and 4 *μ*l RNA template. Diethylpyrocarbonate (DEPC)-treated triple-distilled water was added to a final volume of 50 *μ*l. The PCR program was as follows: reverse transcription at 48°C for 45 min; 94°C for 5 min; then 35 cycles of 94°C for 30 sec, 56°C for 30 sec, 72°C for 30 sec, and a final extension at 72°C for 5 min. The PCR products were identified by 1.5% agarose gel electrophoresis.

### Separation of rat ventricular myocytes

Before isolation, the solutions were saturated with O_2_ for 30 min. The rats were anesthetized with a bolus dose of 20% urethane (6 ml/kg) and fixed on the surgery table. The heart was exposed by rapid thoracotomy, removed and slightly modified in cold (4°C) calcium-free solution. The aorta was fixed to the heart tube of a Langendorff perfusion device with a surgical suture. Then, the isolated heart was perfused for 6 min through the aorta with calcium-free solution and then for 8–12 min with a calcium-free buffer containing 50 *μ*M Ca^2+^, 0.33 g/l collagenase and 0.5 mg/ml bovine serum albumin. The digestion was terminated when the heart became soft and enlarged, followed by washing for 5 min. The entire process was performed while maintaining the perfusion pressure at ∼70 cm at 37°C. Following perfusion, the separated ventricles were cut into small pieces, incubated in a fresh calcium-free solution and mechanically dispersed using a large-bore fire-polished Pasteur pipette. Single intact cells were separated by filtration. Recalcification of the isolated cells was performed using 0.1 mM Ca^2+^ per 10 min to a final calcium concentration of 0.8 mM.

### Recording action potentials (APs) and transient outward potassium current (I_to_)

Only quiescent rod-shaped cells that presented clear cross-striations were selected. All recordings were obtained at room temperature (22–25°C). A small aliquot (∼1.5 ml) of the solution containing the isolated cells was placed in an open perfusion chamber mounted on the stage of an inverted microscope. Cardiac myocytes were allowed to adhere to the bottom of the chamber for 8 min and were then perfused at 1.5 ml/min with Tyrode’s solution.

Electrodes were pulled with a microelectrode puller from borosilicate micropipettes with inner filaments. The resistance of the recording pipette filled with the pipette solution was 3–5 mΩ. The capacitance and series resistance compensation were optimized; 60–80% compensation was usually obtained. The potential compensation was ∼2 mV. The whole-cell configuration of the patch-clamp technique was used to record APs in the current-clamp mode. APs were elicited by stimulation (square wave, 10 msec duration, 100–120% excitation threshold current, 600 pA and 0 mV constant voltage). I_to_ was evoked by depolarizing pulses from a holding potential of −80 mV to voltage steps from −40 to +60 mV in 10 mV increments for 250 msec. The calcium current was blocked by adding 0.1 mM CdCl_2_ to Tyrode’s solution. A prestep of 30 msec to −40 mV was used to inactivate sodium currents. The inactivation of I_to_ was measured after a 400 msec conditioning prepulse at 0.2 stimulation frequency from a holding potential of −80 mV to potentials between −40 and +40 mV (in 10 mV steps), followed by a 250 msec depolarizing pulse to +40 mV at 0.5 stimulation frequency.

### Statistical analysis

SPSS 10.0 (SPSS, Inc., Chicago, IL, USA) was used to analyze the data. All values are expressed as mean ± standard deviation (SD). The significance of differences among groups was determined by single factor analysis of variance (ANOVA). The least significant difference test was applied for multiple comparisons between groups. A t-test was used to compare the significant differences between the pre- and post-experiments. P<0.05 was considered to indicate a statistically significant difference.

## Results

### Changes of rat SBP

**Figure 1.**
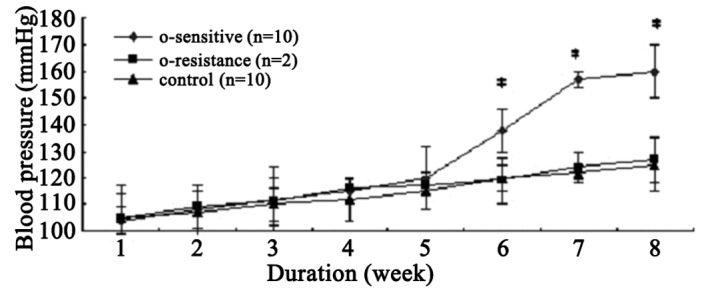
Effects of ouabain on rat systolic blood pressure.

The effects of the continued infusion of EO on the SBP in the first 4 weeks were examined. The SBP of all rats slightly increased and was accompanied by increased weight. However, no significant differences were observed between the O and N groups. During the fifth week, the SBP of 10 of the 12 rats in the O group began to increase. After 6 weeks, the SBP of the 10 rats was significantly higher compared with that in the N group, in which the rats received normal saline (138.2±8.0 vs. 120.1±5.2 mmHg; P<0.01). The 10 rats were designated as the OS rats. The remaining two rats in the O group, with normal SBP after 8 weeks of treatment (126.7±11.4 vs. 125.4±6.9 mmHg; P>0.05) were designated as the OR rats (Fig. 1).

### Ultrastructural changes in the rat myocardium

**Figure 2.**
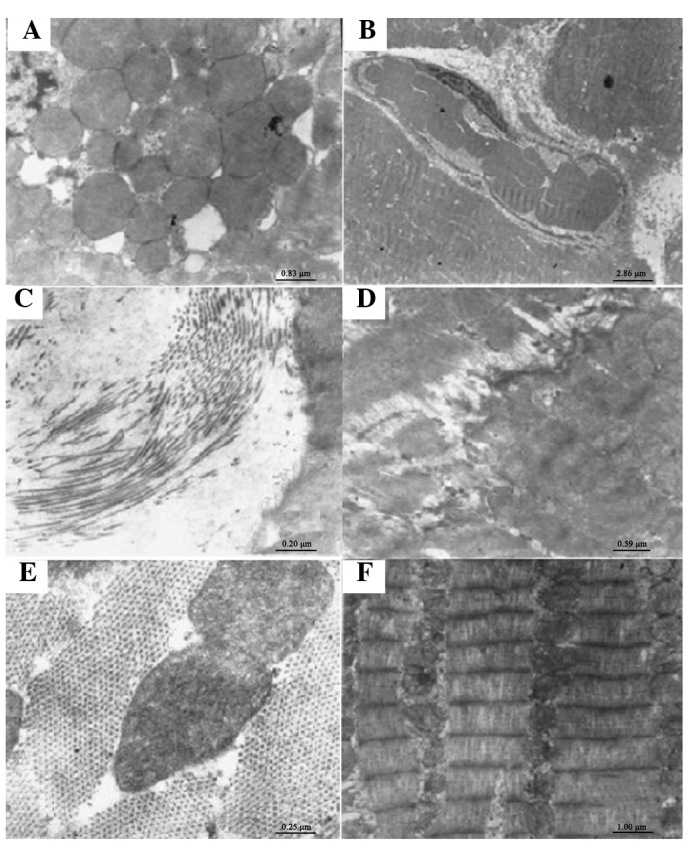
Ultrastructural changes of the rat myocardium. (A) Mitochondrial proliferation, hypertrophy and swelling of the cardiomyocytes of the ouabain-sensitive (OS) rats (magnification, ×12,000). (B) Microvascular membrane permeability in myocardial tissue and exudation around the vessels increased in the OS rats (magnification, ×3,500). (C) Proliferation of collagen fibers among myocardial cells in the OS rats (magnification, ×50,000). (D) Connections of the intercalated discs between adjacent myocardial cells were unclear in the OS rats (magnification, ×17,000). (E) No significant difference was observed in the ultrastructural changes of the myocardium between the ouabain-resistant group and the control group (magnification, ×40,000). (F) The morphology of myocardial cells in the control group was intact. The thick and thin myofilaments were arranged regularly. The uniformly sized mitochondria were abundant and had a round or oval shape. Sarcomeres and light-dark bands were clearly visible. The peri-cellular membrane was uninterrupted and intact (magnification, ×10,000).

The pathological changes in the left ventricular apical mid-myocardium of the OS rats were observed under electron microscopy. The cardiac mitochondria were hyperplastic, hypertrophic and swollen (Fig. 2A). The microvascular membrane permeability in the myocardial tissue and exudation around the vessels increased (Fig. 2B). Collagen fibers proliferated among the myocardial cells (Fig. 2C). The connections of the intercalated discs between adjacent myocardial cells were indistinct (Fig. 2D). There was no significant difference between the ultrastructural changes in the myocardium of rats in the OR and N groups (Fig. 2E).

The left ventricular myocytes in the N group were regularly arranged. The morphology of the myocardial cells was intact. The thick and thin myofilaments were regularly arranged. Uniformly sized, oval mitochondria were abundant. Sarcomeres and light-dark bands were clearly visible. The pericellular membrane was uninterrupted and intact. There was a small amount of collagen fibers among the myocardial cells (Fig. 2F).

### K_V_4.2 expression

There were no significant differences between the K_V_4.2 expression levels in the myocardial cells of the OS (51,345±10,230), OR (53,000±9,880) and control rats (49,878±12,540) (P>0.05).

### APs and I_to_

**Figure 3.**
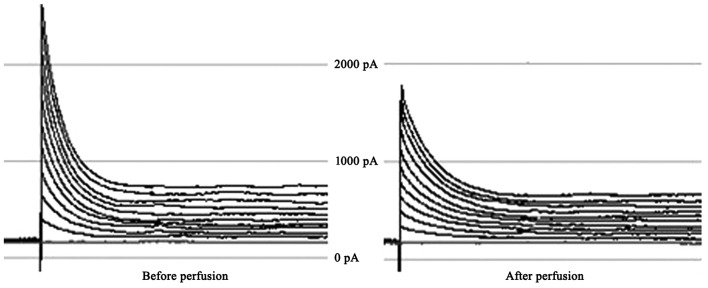
Changes of action potential duration (APD) of rat cardiomyocytes perfused with ouabain (cell count, n=15).

**Figure 4.**
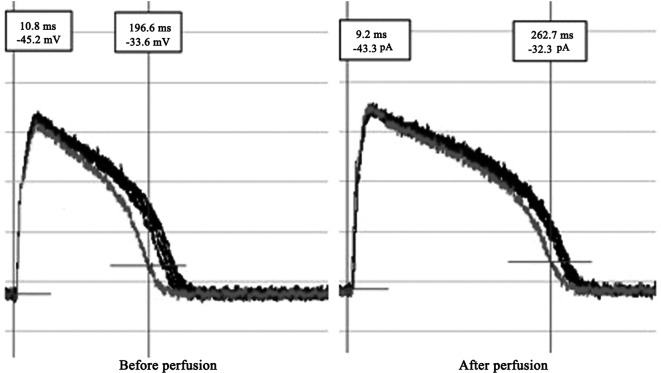
Changes of transient outward potassium current (I_to_) of rat cardiomyocytes perfused with ouabain (cell count, n=15).

APs were recorded under the whole-cell current clamp configuration in rat ventricular myocytes. The AP duration (APD) of the cardiomyocytes was prolonged after they were perfused for 1 min with 1.8 nmol/l EO. The difference was statistically significant; the APD at 50% amplitude (APD50) before and after perfusion was 183±21 and 252±30 msec, respectively (P<0.01). I_to_ of the current density significantly decreased (I_to_ values before and after perfusion were 2,400±231 and 183±104 pA, respectively; P<0.01; cell count, n=15; Figs. 3 and 4).

**Figure 5.**
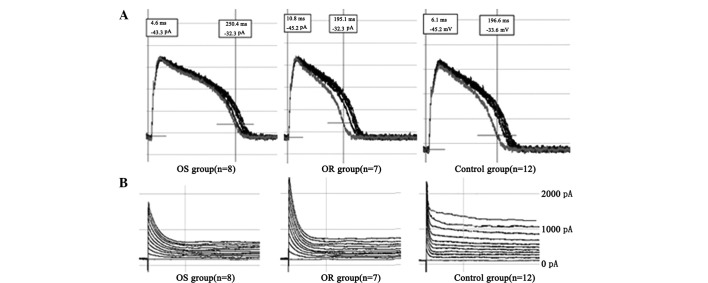
(A) Changes of rat ventricular myocyte action potentials in the ouabain-sensitive (OS), ouabain-resistant (OR) and control (N) groups. (B) Transient outward potassium current (I_to_) changes of rat ventricular myocyte action potentials in the OS, OR and control groups.

The recordings of the changes in AP and I_to_ values of the rat ventricular myocytes in certain models and the N group indicated significant differences between the APD_90_ values of the OS group (242±23 msec; cell count, n=8) and N group (188±31 msec; cell count, n=12; P<0.01). No significant differences were observed between the APD_90_ values of the OR group (184±19 msec; cell count, n=7) and N group (188±31 msec; cell count, n=12; P>0.05). As shown in Fig. 5A and B, the I_to_ of rat ventricular myocytes in the OS group (1,854±192 pA; cell count, n=8) was significantly increased compared with that in the control group (2,413±331 pA; cell count, n=12; P<0.01). No significant difference was observed between the I_to_ of the OR group (2,580±231 pA; cell count, n=7) and N group (2,413±331 pA; cell count, n=12; P>0.05).

## Discussion

EO, a steroidal hormone, is synthesized by the hypothalamus and adrenal cortex (1). EO participates in the neuroendocrine regulation of the cardiovascular system by inhibiting the sodium pump on the cell membrane. Previous studies indicated that EO levels are closely related to blood pressure and cardiovascular complications, including heart failure in rats and humans, during early hypertension (13–16). EO also affects hemodynamics. Intravenous application of EO produced an excitatory effect on baroreceptor nerve activity that was greater in hypertensive rats (17). Exposure of rats to nanomolar concentrations of EO for a longer period of time leads to arterial hypertension (18,19) and smooth muscle proliferation (20,21). In patients with essential hypertension, the circulating endogenous EO concentration correlates directly with the relative wall thickness of the heart and the total peripheral resistance; however, it is inversely correlated with the left ventricular end-diastolic index, stroke index and cardiac index (6).

EO also causes changes in the general morphology of the left ventricle in patients with essential hypertension (6,22). In addition, several reports, including the present study, have also demonstrated that there is a correlation between EO and endothelial dysfunction (12,23,24). In the present study, certain significant pathological ultrastructural changes were also identified in rat apical mid-myocardium, in accordance with the findings of a previous study (25). We successfully constructed an experimental animal model of hypertension using intraperitoneal EO injection. The blood pressures of the majority of rats (10 out of 12 rats) increased following injection of EO. The remaining two rats in the O group that did not have increased SBP after 8 weeks of treatment were designated as OR rats. Clear pathological changes were observed in the left ventricular apical mid-myocardium of the OS rats under electron microscopy. The changes included the rupture of the aortic intima and outer membranes, as well as endothelial cell loss. Although no significant loss of the capillary endothelial cells in the myocardium was observed, microvascular membrane permeability in the myocardial tissue and exudation around the vessels increased. These pathological changes in the endothelial cells from the main artery to capillaries may lead to the weakening or disappearance of functional membrane barriers, thereby initiating the remodeling of hypertensive vessels. The structural integrity of the mitochondria is important for maintaining the cellular structure. Proliferation and swelling of the mitochondria in the myocardial cells were observed. The EO injection also caused structural damage to the intercalated discs between adjacent myocardial cells. The proliferation of collagen fibers among myocardial cells was also observed under electron microscopy. Myocardial fibrosis caused by increased synthesis of collagen fibers is an important pathological basis for ventricular remodeling during hypertension, resulting in systolic and diastolic dysfunction.

Previous *in vitro* studies suggested that the function of EO in vascular endothelial cells is related to the maintenance of reproductive metabolism in normal endothelial cells under physiological concentrations. EO at pathological concentrations may induce damage to human vascular endothelial cells, resulting in direct endothelial injury and the initiation of hypertensive vascular remodeling (1,12,13). The consequential target organ damage is also aggravated.

We investigated the effects of EO on myocardial electrical remodeling *in vitro* and *in vivo*. The results of isolated perfusion with EO and the direct study of EO in ventricular myocytes in hypertensive rats revealed that EO causes prolonged APs and reduced I_to_ density. The results suggested that EO may cause increased blood pressure and lead to cardiac remodeling. Consequently, the proliferation of collagen fibers among myocardial cells, mitochondrial proliferation and swelling in myocardial cells, as well as the deposition of glycogen were observed under electron microscopy. However, EO may also lead to myocardial electrical remodeling due to the direct effect on myocardial electrophysiology, resulting in prolonged APs and decreased I_to_ density. Previous studies demonstrated that a number of pathological conditions, including myocardial ischemia, stress overloading, neurohumoral factors and heart failure, lead to reduced I_to_ density and cardiac electrical remodeling (26–29). In the present study, the results of real-time RT-PCR indicated no significant difference in K_V_4.2 expression levels in the ventricular tissues of the three groups of animal models. This finding suggests that, although there was no significant difference among the transcriptional levels of the three groups, the slightly varied levels may be caused by discrepancies in the translation and functional protein levels due to certain factors. The specific mechanism remains unclear.

In view of this, and in line with our previous findings, it is possible that EO may participate in the cardiac structural and electrical remodeling of hypertension (30,31). Therefore, a significant neuroendocrine hormone, such as EO, may be used as a target for the clinical treatment of hypertension to reduce blood pressure and myocardial remodeling (16).

## Figures and Tables

**Table I.** Primer sequences of K_v_4.2 and GAPDH

Gene	Primer sequence
K_v_4.2	Forward: 5-GCCTTCGTTAGCAAATCTGGATC-3
	Reverse: 5-CACTTCCATGCAGCT TTCTTCAA-3
	Probe: 5-FAM-CGAGACAACACCACCACCTGCTTCACTA-MRA-3
GAPDH	Forward: 5-TGGTCTACATGTTCCAGTATGACT-3
	Reverse: 5-CGTTTGATGTTAGCGGGATCTC-3
	Probe: 5-FAM-ACGGCAAGTTCAACGGCACGTCAATA-MRA-3

K_V_4.2, voltage-gated potassium channel 4.2; GAPDH, glyceraldehyde-3-phosphate dehydrogenase.
